# Cholecystectomy with the Hugo™ robotic-assisted surgery system: the first general surgery clinical study in Korea

**DOI:** 10.1007/s00464-024-11334-4

**Published:** 2024-10-28

**Authors:** Wooil Kwon, Jin-Young Jang, Chang Wook Jeong, Sylvain Anselme, Fabio Pradella, Jacklyn Woods

**Affiliations:** 1 Department of General Surgery, Seoul National University Hospital, Seoul, Republic of Korea; 2 Department of Urology, Seoul National University Hospital, Seoul, Republic of Korea; 3 Clinical & Regulatory Solutions, Medtronic Inc., Rome, Italy; 4 Surgical Robotics, Medtronic Inc., 710 Medtronic Pkwy NE, Minneapolis, MN 55432 USA

**Keywords:** Robotic-assisted surgery, Cholecystectomy, Novel robotic systems, Robotic cholecystectomy, Emergent robotic systems

## Abstract

**Background:**

The Hugo™ Robotic-Assisted Surgery (RAS) System is an emergent device in the robotic surgery field. This study aims to describe the first general surgery-focused clinical study in Korea using the novel Hugo™ RAS System.

**Methods:**

This study was a prospective, single-center, single-arm, confirmatory clinical study conducted at Seoul National University Hospital where 20 cholecystectomies were performed. To evaluate the safety and performance of the Hugo™ RAS System the incidence of conversion to laparoscopy or open surgery, major complication (Clavien-Dindo Grade ≥ III) rate, overall complication rate, readmission rate, and reoperation rate were evaluated. All parameters were assessed within 30 days post-procedure. Any device deficiencies encountered during our initial experience and device data such as setup, console, and operative times were also reported.

**Results:**

We confirmed that our trial achieved the primary objective with a success rate of at least 95%. This was accomplished with no conversions to other types of surgery due to serious system malfunction and with only one major complication within 24 h post-procedure. The 20 consecutively enrolled patients had a median age and BMI of 58 years old and 23.9 kg/m^2^, respectively. The major complication rate was 10% (2/20 patients), the overall complication rate was 15% (3/20 patients), the readmission rate was 15% (3/20 patients), and the reoperation rate was 0% (0/20 patients). None of the complications were definitively device related. The median setup, console, and operative times were 16, 17, and 55 min, respectively. The device deficiency rate was 15% (3/20 patients), but all device deficiencies were minor, occurred before the first incision, and did not present a risk to the patient.

**Conclusion:**

Based on our initial experience with the Hugo™ RAS System, cholecystectomy is feasible and safe. This trial is registered with ClinicalTrials.gov (NCT05715827).

Though the prevalence of gallstone disease shows geographic and ethnicity-based differences, it is a common digestive disease affecting 10% to 15% of the global population [1]. In Europe, the rate of gallstone disease increases with age and in every age range there is an increased prevalence in women rather than men [2, 3]. Regional disparities within Europe are most likely due to variations in diet and obesity rates [2]. In the United States, a region with a unique diet and a high obesity rate, 20 million adults are affected by gallstone disease at an approximate cost of $6.2 billion [3]. Yet, even in regions with a low obesity rate, such as Eastern Asia, the incidence and prevalence of gallbladder disease are still fairly high [3]. Nonetheless, the manifestation of gallstone disease is typically different in Asian countries. Compared to Western populations, East Asians have a higher prevalence of pigmented stones and stones located in the common bile duct [4]. In the Republic of Korea, hereafter referred to solely as Korea, a recent study showed the incidence of surgeries to treat gallstone disease has steadily increased over the last decade [5].

Laparoscopic cholecystectomy is the current standard of care for the treatment of gallstone disease [6, 7]. Even so, the frequency of robotic cholecystectomy has increased each year since 2017 [8]. When examined via meta-analysis, robotic cholecystectomy had a higher operative time compared to laparoscopic cholecystectomy, but the safety and perioperative outcomes were similar [9]. For robotic cholecystectomy to continue to gain acceptance in the field, there would need to be equipment benefits to the surgeons that still maintained the standard of care for the patients.

The da Vinci surgical system has been the leader in robotic surgery for the past two decades. However, their patent recently expired and several manufacturers worldwide have sought to develop their own surgical robotic platforms. A relatively new device on the market is the Hugo™ Robotic-Assisted Surgery (RAS) System (Medtronic Plc, Dublin, Ireland). The Hugo™ RAS System was introduced to the European market in March 2022 with CE (Conformité Européenne) approval for urology and gynecology, followed by general surgery in October 2022. This platform consists of a system tower, four independent arm carts, and an open surgeon console. The goal of this new modular system was to offer equipment benefits that are necessary for robotic surgery to continue to increase in use for general surgery procedures, while still operating with a comparable safety profile for patients.

This pre-market study represents the first general surgery procedures conducted in Korea using the Hugo™ RAS System. The data presented in this study are a subsection of the data collected for a larger multi-indication investigational study conducted in Korea, entitled Hello Hugo. The aim of this study was to evaluate the safety and effectiveness of cholecystectomy with the first generation of the Hugo™ RAS System.

## Methods

### Study design

This study was a prospective, single-center, single-arm, pivotal clinical trial to evaluate the feasibility of cholecystectomy with the Medtronic Hugo™ RAS System. The presentation of these data represents a subset of a larger study. The other indication from the Hello Hugo pre-market investigational study was focused on urology and is currently under review to be published. The Hugo™ RAS System has received approval from the Ministry of Food and Drug Safety (MFDS) of Korea in February 2024. The full study for the MFDS is registered on ClinicalTrials.gov (http://clinicaltrials.gov; NCT05715827).

This study was conducted in compliance with the Declaration of Helsinki, Good Clinical Practice, ISO 14155:2020 requirements, the Medical Device Act, the Personal Information Protection Act, the Korea Medical Devices Industry Association Fair Trade Competition, and the site’s Institutional Review Board (IRB) requirements.

The principles of the Declaration of Helsinki were implemented through the informed consent process, IRB approval, study training, clinical trial registration, pre-clinical testing, risk–benefit assessment and publication policy. The sponsor of this trial, Medtronic, ensured to avoid improper influence on or inducement of the patients, monitors, investigators or other parties participating in or contributing to the clinical investigation.

### Patients

A total of 22 cholecystectomy-indicated patients provided written consent and were enrolled consecutively in the study from February to April 2023. However, two cholecystectomy patients exited the study prior to surgery due to a surgeon decision to undergo laparoscopic surgery due to severe adhesion and inflammation for one patient and a console communication error prior to the first incision for the other. Instead, these two patients underwent a laparoscopic procedure. Thus, the remaining 20 cholecystectomy-indicated patients underwent a procedure with the Hugo™ RAS System and completed their corresponding post-procedure 30-day follow-up visit.

Inclusion criteria were patients indicated for a cholecystectomy with cholelithiasis, cholecystitis, or gallbladder polyps. Specifically, gallbladder polyps had to be ≥ 10 mm, enlarging, or symptomatic.

Exclusion criteria were: patients with a considerable risk for laparoscopic surgery (e.g., severe cardiopulmonary diseases which contraindicated to general anesthesia, uncontrolled coagulopathy, etc.), patients requiring urgent surgery, pregnant or lactating women, patients with severe liver cirrhosis (Child–Pugh class C) with portal hypertension or suspicion of gallbladder cancer, previous abdominal surgery within the past two years, concurrent participation in another clinical study, patients with a condition that could compromise study compliance (e.g., mentally incompetent, alcohol or drug abuse) as determined by the investigator, and patients who were considered unsuitable to conduct the trial as determined by the investigator.

### Outcome measures

Major complications were defined as any complication with a Clavien-Dindo Grade ≥ III. The primary study objective was a composite of a completed cholecystectomy with the Hugo™ RAS System without conversion (to open or laparoscopic surgery), and no major complications within 24 h post-procedure. The primary objective of the trial was considered achieved if the composite rate was at least 95%.

The secondary study endpoints assessed the short-term safety outcomes via the: major complication rate, overall complication rate, readmission rate, reoperation rate, and device deficiency rate all through 30 days post-procedure.

The other clinical data collected for this study subset were: baseline patient characteristics (e.g., age, height, weight, etc.), pathology characteristics, estimated blood loss, surgical setup time, total console time, operative time, and robot take-down time.

### Complications

A complication in this study was defined as an unanticipated problem that arose following and was a result of, a procedure, treatment, or illness. This definition is aligned with the adverse events definition (ISO 14155:2020). Expected adverse events were not captured or reported unless the adverse event worsened or persisted. Expected adverse events included: anesthesia-related nausea/vomiting, low-grade fever (< 37.8°C), incisional pain, sleep problems (insomnia), constipation, and mild to moderate bruising/ecchymosis. The relationship of a complication to the device, underlying condition, surgical procedure, or anatomical factor was determined by the site.

### Hugo™ RAS system

The Medtronic Hugo™ RAS System is a modular robotic platform for performing robotic-assisted minimally invasive surgery. The Hugo™ RAS System has three main components: a system tower, movable individual arm carts, and an open surgeon console.

**Fig. 1:**
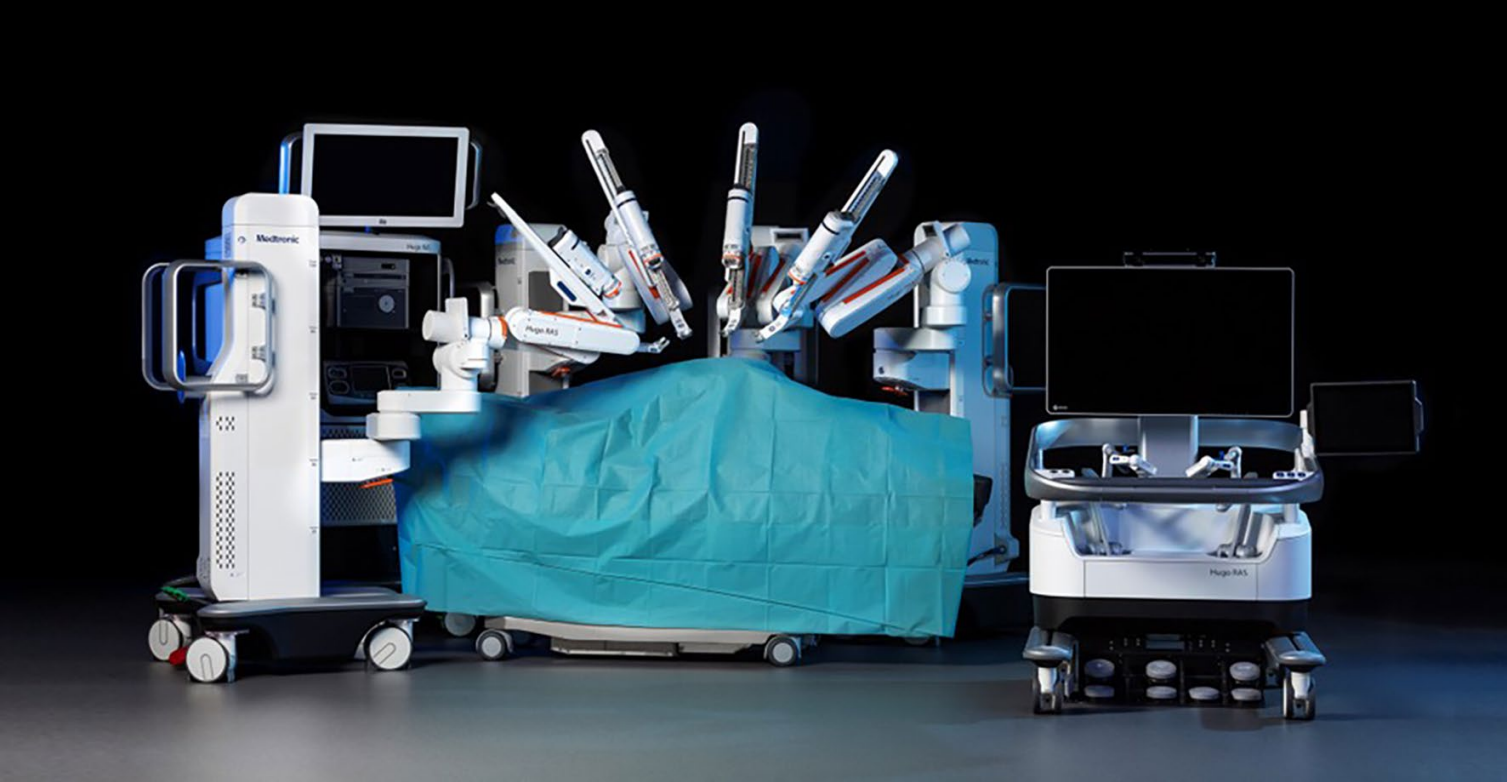
The Hugo™ RAS System Overview System tower, modular arm carts, surgeon console

The system tower houses computers, the endoscope system, the electrosurgical generator, the power management system with a backup battery, and a high-definition interactive touchscreen display. The arm carts consist of movable platforms with casters which support an extendable robotic arm. Up to four arm carts can be connected to the system tower for simultaneous use during surgery. The arm carts are movable within the operating room (OR) and the hospital. Prior to surgery, the OR team positions the arm carts around the surgical table according to the surgical procedure. The team can adjust the arm carts and extendable arms to accommodate patient positioning and optimize bedside access to the patient. The surgeon console is an open console that consists of a flat screen with a high-definition three-dimensional (3D) display, a touchscreen interactive display, adjustable ergonomic controls, an armrest, two surgeon hand controllers, a set of foot pedals, and 3D surgeon and observer glasses. The surgeon hand controllers respond to wrist movement. Sensors in the surgeon console track the movement of the 3D glasses worn by the surgeon and can clutch the movement of the instruments if the surgeon looks away from the 3D display (Fig. 1).

### Surgical setup

**Fig. 2:**
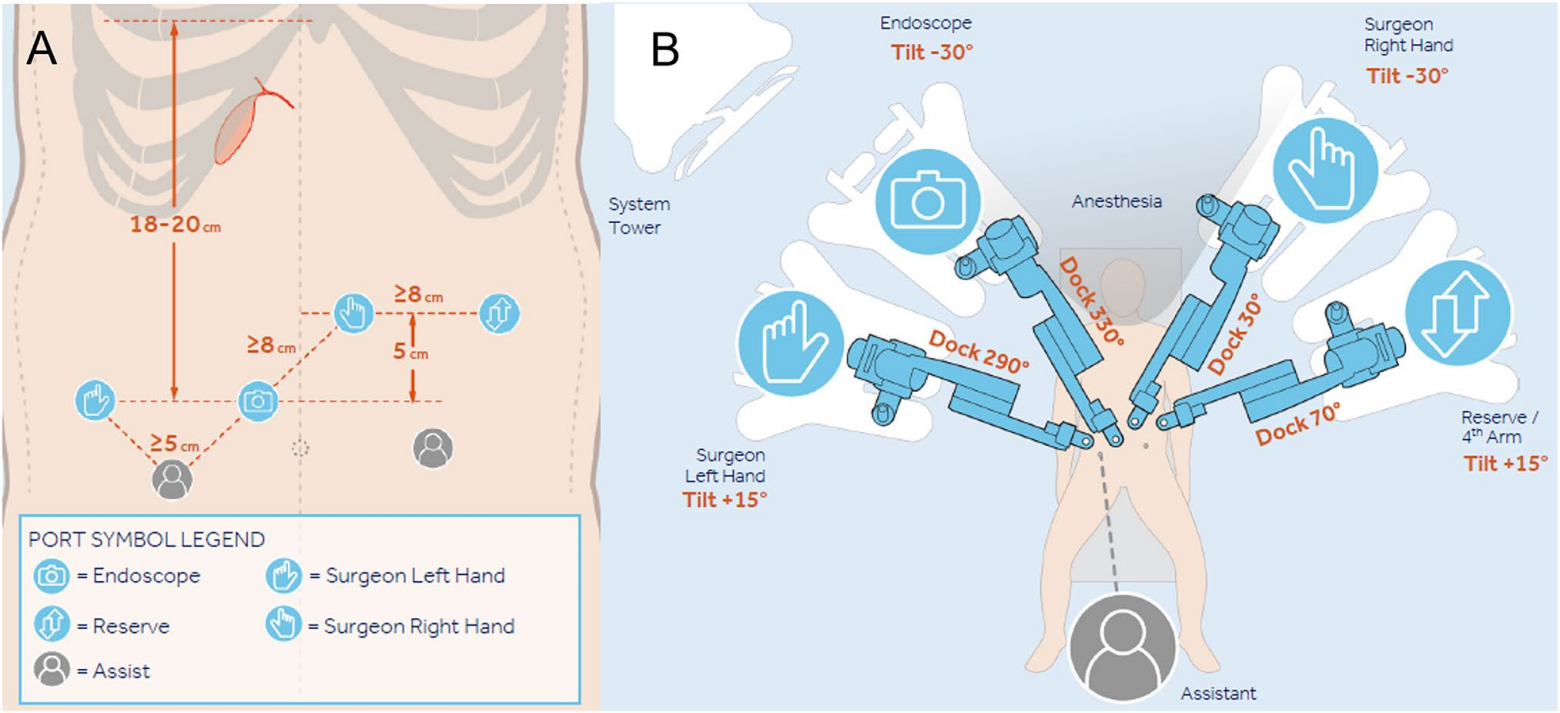
Surgical Setup of the Hugo™ RAS System **A** Port placement and **B** Arm cart positioning for cholecystectomy procedures

All patients were positioned in reverse Trendelenburg (> 20°) under general anesthesia. Incisions for ports were made according to the Medtronic Setup Guide and slightly adjusted to minimize the number of incisions. The camera port was placed through a periumbilical incision. The remaining ports were placed at a safe distance from the camera port and adjacent ports (Fig. 2A). The robotic arms were positioned according to the Medtronic Setup Guide (Fig. 2B). The endoscope and wristed instruments were attached to the robotic arms and inserted into the ports.

### Surgical procedure

After identification of the gallbladder, the fundus was retracted cephalad over the liver with a grasping instrument. Adhesions were taken down using the Maryland dissector. An additional grasper was used to retract the gallbladder inferolaterally to expose the triangle of Calot. The separation of the cystic duct anteriorly from the cystic artery was performed with a Maryland grasper. The cystic artery was clipped and then divided by hook scissors. The dissection of the cystic pedicle was completed by placement of a clip to occlude the cystic duct. The gallbladder was separated from the liver with scissors with an electrosurgical attachment or a monopolar hook. After the gallbladder resection was complete, the gallbladder was placed in a retrieval bag and removed through the umbilical port. All incised port sites were closed.

### Statistical analysis

Descriptive statistics were used to present the data and to summarize the results. Continuous variables were summarized with number of patients (n), mean, standard deviation (SD), median, and ranges. Categorical variables were summarized by frequencies and percentages.

To estimate the sample size, two things were taken into account: the number of patients from previous studies [10], and the minimum number to show whether the primary objective was met [11]. Based on these two considerations, the planned study population was 20 cholecystectomy patients.

## Results

A total of 20 cholecystectomy procedures were performed between February and April 2023 using the Hugo™ RAS System. The median patient age was 58 (range 27–71) years old and 60% of the patients were female. The median BMI was 23.9 (range 19–31) kg/m^2^. For an assessment of the patients’ overall health, the American Society of Anesthesiologists (ASA) classification system was used. Of the 20 total cholecystectomy patients, five had an ASA class of I, 14 had an ASA class of II, and one had an ASA class of III (Table 1).

**Table 1:** Characteristics of the study population

Patient characteristics	(N = 20 patients)
Age (years)	
Mean (SD)	56.5 (10.6)
Median	58.0
Min, Max	27, 71
Sex, % (n/N)	
Male	40.0% (8/20)
Female	60.0% (12/20)
Weight (kg)	
Mean (SD)	65.6 (11.6)
Median	65.1
Min, Max	49, 87
Height (cm)	
Mean (SD)	163.4 (6.5)
Median	161.9
Min, Max	154, 175
BMI (kg/m^2^)	
Mean (SD)	24.5 (3.3)
Median	23.9
Min, Max	19, 31
ASA Physical Status Classification, % (n/N)	
ASA I—A normal healthy patient	25.0% (5/20)
ASA II—A patient with mild systemic disease	70.0% (14/20)
ASA III—A patient with severe systemic disease	5.0% (1/20)
ASA IV—A patient with severe systemic disease that is a constant threat to life	0.0% (0/20)
ASA V—A moribund patient who is not expected to survive without the operation	0.0% (0/20)
ASA VI—A declared brain-dead patient whose organs are being removed for donor purposes	0.0% (0/20)

Setup time was defined as the sum of the time to complete the following: plug in and calibrate all robotic arms, drape all robotic arms with a sterile drape, position all robotic arms around the operating table, and connect all robotic arms to the ports. The median setup time for the 20 cholecystectomy procedures was 16 (range 10–26) minutes. Console time was defined as the amount of time the surgeon spent at the Hugo™ RAS surgeon console during the procedure. The median console time was 17 (range 12–58) minutes. Operative time was defined as the total length of the procedure. The median operative time was 55 (range 35–123) minutes. The take-down time was defined as the sum of the time to complete the following: disconnect the robotic arms from the ports, move the robotic arms from around the operating table, and position the robotic arms to a location conducive to storage. The median take-down time was 3 (range 1–6) minutes (Table 2).

**Table 2:** Surgical procedure details

Surgical procedure details	(N = 20 Patients)
Setup time, min	
Mean (SD)	17.8 (4.9)
Median	16.0
Min, Max	10, 26
Total console time, min	
Mean (SD)	19.8 (10.2)
Median	17.0
Min, Max	12, 58
Operative time, min	
Mean (SD)	58.5 (18.9)
Median	55.0
Min, Max	35, 123
Robot take-down time, min	
Mean (SD)	3.1 (1.1)
Median	3.0
Min, Max	1, 6
Estimated blood loss, mL	
Mean (SD)	1.3 (2.4)
Median	0.0
Min, Max	0, 10
Blood transfusion during surgery	0.0% (0/20)
Use of drainage tube	
Yes	0.0% (0/20)
No	100.0% (20/20)

The median estimated blood loss during the 20 cholecystectomy procedures was 0 (range 0–10) mL. The median length of hospital stay was 1 (range 1–3) day. A drainage tube was not used in any of the patients (Table 2). Other measurements such as quantity and location of gallbladder stones, polyps, and adhesions were also collected (Table 3).

**Table 3:** Additional measurements

Post procedure details	(N = 20 patients)
Acute cholecystitis	
Yes	10.0% (2/20)
No	90.0% (18/20)
Gallbladder stones	
Yes	75.0% (15/20)
No	25.0% (5/20)
Gallbladder polypoid lesions	
Yes	50.0% (10/20)
Adenomyomatosis	25.0% (5/20)
Cholesterol polyps	25.0% (5/20)
Amount of gallbladder adhesions	
No adhesions	55.0% (11/20)
Adhesions < 50% of gallbladder	35.0% (7/20)
Adhesions burying gallbladder	10.0% (2/20)

The primary study objective was a composite of a completed cholecystectomy with the Hugo™ RAS System without conversion and no major complications within 24 h post-procedure. There were no conversions to open or laparoscopic surgery from the Hugo™ RAS System. There was one major complication within 24 h of the cholecystectomy procedure (Table 4).

**Table 4:** Primary and secondary objective of the study

Primary objective characteristics	% (n/N)
Composite completion rate	95.0% (19/20)
Rate of conversions to open or laparoscopic surgery	0.0% (0/20)
Rate of major complication within 24 h	5.0% (1/20)

Secondary objective characteristics	n of events (n/N, % of patients)
Overall complications (All Grades)	7 (3/20, 15.0%)
Complication by Clavien-Dindo Grade	
Grade I	1 (1/20, 5.0%)
Grade II	3 (2/20, 10.0%)
Grade IIIa	2 (1/20, 5.0%)
Grade IIIb	1 (1/20, 5.0%)
Grade IV	0 (0/20, 0.0%)
Grade V	0 (0/20, 0.0%)
Major complications (through 30 days)	3 (2/20, 10.0%)
Readmission rate (through 30 days)	4 (3/20, 15.0%)
Reoperation rate (through 30 days)	0 (0/20, 0.0%)
Device deficiency rate (through 30 days)	3 (3/20, 15.0%)

The secondary study endpoints assessed the major complication rate, overall complication rate, readmission rate, reoperation rate, and device deficiency rate through 30 days post-procedure. A total of seven complications were reported in three of the 20 patients in this study through 30 days post-procedure. Of the seven complications, one was Grade I, three were Grade II, two were Grade IIIa, and one was Grade IIIb (Table 4).

The Grade IIIb complication occurred within 24 h of the procedure and was a small intestinal perforation (1 cm) during the umbilicus incision that was repaired during the primary procedure. The patient recovered, was given fluids, and a prolonged hospital stay of three days. The complication was caused by the surgical procedure and anatomical factors. The Grade IIIa complications occurred within 30 days post-procedure and were a cystic duct remnant common bile duct stone requiring endoscopic retrograde cholangiopancreatographic (ERCP) stone removal and a minor cystic duct leakage that occurred after the ERCP procedure. Both complications occurred in the same patient and both complications resolved, though the patient did have to be readmitted. These complications were potentially caused by an underlying condition and the endoscopic procedure. The Grade II complications occurred within 30 days post-procedure and were vomiting, hypertension, and a mid-abdominal wall hematoma. The patient recovered from vomiting after being readmitted. This complication was potentially caused by anatomical factors. The same patient experienced two complications, hypertension and a subcutaneous hematoma adjacent to the port incision site. This patient recovered but was also readmitted for the hematoma and given supportive care with pain control and antibiotics. The hematoma was judged to potentially be caused by the procedure or the device. The hypertension was judged to be caused by the procedure. The Grade I complication occurred within 24 h of the procedure and was seroma discharge from the port site. The patient recovered and the complication was judged to potentially be related to the procedure. None of the complications were definitively caused by the Hugo™ RAS System. All patients recovered from their complications and no deaths were reported during this study.

The readmission rate was 15% (3/20) through 30 days post-procedure. No reoperations occurred during this study. There were three device deficiencies reported during three patients’ procedures (Table 4). All of these device deficiencies were related to the arm carts. All errors occurred during setup and preparation prior to surgery. The OR staff followed the description for resolution in the User’s Guide, such as recalibrating the arm and clearing the error message. All arm cart deficiencies were resolved without further incidence and no possibility of a serious adverse device effect (SADE) or possibility of harm to the patient.

## Discussion

This is the first general surgery-focused study conducted in Korea using the Hugo™ RAS System for 20 cholecystectomies. The composite primary objective of this study was achieved with no conversions to open or laparoscopic surgery and only one major complication in the 24 h post-procedure. Overall, seven complications were reported in three patients through 30 days post-procedure. All patients recovered from their complications and none of those complications were definitively device related. The readmission rate was low and no reoperations were necessary during this study. Three minor device deficiencies were reported, but all occurred before the first incision and were quickly remedied. None of the device deficiencies were judged to potentially cause a SADE. The clinical outcomes from this cholecystectomy-focused study contribute to the growing global data on the feasibility of performing general surgery procedures with the Hugo™ RAS System.

Using the Hugo™ RAS System our cholecystectomy data were comparable to published data using other robotic systems, as well as the current standard of care, laparoscopic surgery. In terms of perioperative outcomes our median operative time was 55 (range 35–123) minutes, which was less than others’ initial experiences with emergent robotic platforms. For example, the Senhance system had a median operative time of 86.5 (interquartile range [IQR] 60.5–106.5) [12] and 71.5 (range 34- 197) minutes [13], and Revo-i had a mean time of 115 (SD ± 17.3) minutes [10]. Our operative time was also less than the well-established robotic platform of da Vinci Si, with a mean operative time of 92.7 (SD ± 22.7) [14]. One of the Senhance studies also performed 20 laparoscopic cholecystectomies for comparison and reported a median operative time of 31.5 (IQR 26–41) minutes [12]. Though our median operative time with a robotic platform was quite quick, several review articles and registry databases have previously shown that laparoscopic surgery is traditionally faster than robotic surgery [15]. However, many retrospective laparoscopic studies lack that level of granularity for an extensive study-to-study comparison [16]. The median console time using the Hugo™ RAS System was 17 (range 12–58) minutes, which was less than Senhance with a median of 30.8 (IQR 23.5–35) minutes [12], Revo-i with a mean time of 49.7 (SD ± 15.4) minutes [10], and the mean for da Vinci Si of 48.7 (SD ± 23.7) minutes [14]. Our median docking time was 16 (range 10–26) minutes, which was more than Senhance with a median of 11.5 (IQR 9–13) minutes [12] and Revo-i with a mean of 10.6 (SD ± 3.2) minutes [10].

The available data of others using the Hugo™ RAS System for cholecystectomy is limited, but we were able to compare our results to the only other studies currently published. Our study reported median operative, console, and setup times of 55 (range 35–123), 17 (range 12–58), and 16 (range 10–26) minutes, respectively. Caputo et al. reported a median operative, console, and docking time of 100 (range 88–105), 54 (range 38–60), and 10 (range 6–12) minutes, respectively [17]. Belyaev et al. provided the individual times for each of their 14 patients but their console time ranged from 29 to 172 min and their docking time ranged from 5 to 15 min [18]. Vicente et al. published a case report on just one patient and stated an operative time of 70 min and a docking time of 3 min [19]. Though the operative and console times were notably less in our study compared to others, their docking times were faster than our setup time. These discrepancies could be due to slight variations in how each term is defined, as well as the learning curve associated with the first generation of a new system.

In terms of post-operative outcomes, our overall complication rate and major complication rate through 30 days were analogous to others’ experiences with robotic platforms and laparoscopic surgery. Our study reported seven overall complications in three patients, with three of those complications being considered a major complication in two patients using a more strict than usual definition of evaluating safety events. Accordingly, the overall complication rate was 15.0% and the major complication rate was 10%. In the Senhance study where both robotic surgery and laparoscopic surgery were performed, the robotic system had an overall adverse event rate of 30% and none of those were major complications [12]. The laparoscopic cholecystectomies had an overall adverse event rate of 25.0% and 5% were major complications [12]. Other Senhance studies reported a 0% overall complication rate, yet these studies were retrospective [13, 20]. The Revo-i study stated a 0% overall complication rate [10] as well, but the da Vinci Si study had a 3.7% complication rate [14]. None of the other Hugo™ cholecystectomy publications reported complications [17–19]. However, when examining large cross-sectional studies there was a higher rate of complications reported. For example, a study using the National Inpatient Sample showed a complication rate of 14.3% for robotic cholecystectomies, which was slightly less than the 14.6% complication rate of laparoscopic cholecystectomies [21]. Furthermore, a retrospective cohort study using Medicare administrative claims data found that the complication rate for robotic cholecystectomy was 20.5% and laparoscopic cholecystectomy was 20.6% [15].

In order for the use of robotic surgery to continue to increase for general surgery procedures a comparable safety profile to laparoscopic surgery, equipment benefits from the robotic system, and a cost assessment of both should be analyzed. In this study, the Hugo™ RAS System has shown a comparable safety profile to both robotic devices and laparoscopic surgery. The Hugo™ RAS System has the potential to offer equipment benefits with the open surgeon console that is supposed to facilitate OR communication while maintaining an ergonomic position for the surgeon. Yet, further studies are needed to verify this potential. In this investigational study, the Hugo™ RAS System performed well, especially as a first-generation investigational device, with only three device deficiencies. These device deficiencies were related to the arm carts and were identified and rectified before surgery had begun. Lastly, a cost analysis including the Hugo™ RAS System would bolster continued acceptance of robotic cholecystectomy in the field of general surgery and represents an opportunity for future study.

Although this study demonstrated favorable clinical outcomes that speak to the feasibility of using the Hugo™ RAS System for cholecystectomy procedures, we encountered a few limitations. First, a larger sample size would have increased the power of the study. However, the study was adequate for an initial investigation and was aligned with other’s initial studies for the MFDS [10, 22]. Furthermore, this study was a Phase I trial where a relatively small sample size is expected. Phase II trials are being carried out in different geographies. Lastly, though our study exhibited comparability to a variety of RAS devices and laparoscopic surgery studies in an abbreviated cross-section of the literature, a study focused on a direct study-to-study comparison of devices with the same surgeons would be more impactful. All of these limitations are outside the scope of this investigation and represent an opportunity for future studies.

In conclusion, our experience with 20 cholecystectomy patients shows that minimally invasive cholecystectomy with the Hugo™ RAS System is feasible and has a comparable safety profile to other robotic devices, as well as the current standard of care, laparoscopic surgery. Further comparative trials are needed to continue to assess the safety and feasibility of the novel Hugo™ RAS System.
